# P-526. A Low Cost, Low Technology Intervention for Hospitalized Patients Who Inject Drugs is Feasible for Increasing Evidence-Based Addiction Care but HIV PrEP Uptake Remains Limited

**DOI:** 10.1093/ofid/ofae631.725

**Published:** 2025-01-29

**Authors:** Tanvi Bhadkamkar, Kelly Gagnon, Mariel Parman, Kedarius Ingram, Marry Figgat, Stephen O’Rear, William Bradford, Sara Asgari, Ellen Eaton

**Affiliations:** University of Alabama at Birmingham, Birmingham, Alabama; University of Alabama Birmingham, Birmingham, Alabama; University of Alabama at Birmingham, Birmingham, Alabama; University of Alabama at Birmingham, Birmingham, Alabama; University of Alabama at Birmingham, Birmingham, Alabama; University of Alabama at Birmingham, Birmingham, Alabama; University of Alabama at Birmingham, Birmingham, Alabama; University of Alabama at Birmingham, Birmingham, Alabama; University of Alabama, Birmingham, Birmingham, Alabama

## Abstract

**Background:**

Approximately 14% of new HIV cases in Alabama are from injection drug use, and this does not include HIV transmission from substance use related behaviors such as transactional sex. Hospitalized patients who inject drugs (PWID) present an opportunity to provide addiction treatment and HIV pre-exposure prophylaxis (PrEP). The objective of this study is to assess the feasibility and acceptability of novel, low barrier interventions aimed at increasing uptake of medications for opioid use disorders (MOUD) and PrEP in PWID.

**Methods:**

In this prospective study, 60 hospitalized PWID who were HIV negative and receiving care at UAB Hospital (from October 2022 to February 2024) were randomized in a two-by-two factorial design to 4 arms: a smartphrase summary of evidence-based care added in the medical record (n=15), an outpatient peer recovery coach intervention (n=15), both (n=15), or standard of care (n=15). Patients completed standardized surveys on substance use, HIV risk behaviors, and their preferences for MOUD and PrEP at baseline. Exit surveys were conducted by phone to assess utilization of addiction treatment, PrEP services, and peer coaching. Feasibility was primarily defined by the ability to recruit hospitalized PWID and retain them in the study to determine post-discharge clinical outcomes, but feasibility was also defined by appropriate use of smartphrase and access and engagement with the peer. We assessed preliminary effectiveness by including utilization of MOUD, PrEP, and the peer coach.

**Results:**

In the groups who received the smartphrase with or without the peer (n=30), 70% had documentation of the smartphrase in their provider’s consult note, and 90% had a MOUD referenced in their treatment plan. In the groups who received a peer with or without the smartphrase (n=30), 100% were contacted, and 90% had MOUD referenced in treatment plan. However, PrEP was not provided on discharge for any patients in the study.

**Conclusion:**

In this pilot study of two low barrier interventions for PWID, we found that both interventions are feasible and acceptable, but HIV prevention with PrEP is still lacking. Future directions are to expand the sample size to test if these interventions will increase MOUD and PrEP uptake in a Southern cohort of PWID.

**Disclosures:**

**All Authors**: No reported disclosures

